# Double-Clad Fiber-Based Multifunctional Biosensors and Multimodal Bioimaging Systems: Technology and Applications

**DOI:** 10.3390/bios12020090

**Published:** 2022-02-01

**Authors:** Kathy Beaudette, Jiawen Li, Joseph Lamarre, Lucas Majeau, Caroline Boudoux

**Affiliations:** 1 Castor Optics Inc., Montreal, QC H4N 2G6, Canada; jlamarre@castoroptics.com (J.L.); lmajeau@castoroptics.com (L.M.); caroline.boudoux@polymtl.ca (C.B.); 2 Institute for Photonics and Advanced Sensing, School of Electrical Electronic Engineering, The University of Adelaide, Adelaide, SA 5005, Australia; 3 Australian Research Council Centre of Excellence for Nanoscale BioPhotonics, The University of Adelaide, Adelaide, SA 5005, Australia; 4 Department of Engineering Physics, Polytechnique Montreal, Montreal, QC H3T 1J4, Canada

**Keywords:** biosensor, double-clad fiber, fluorescence, multimodal imaging, multi-photon imaging, optical coherence tomography, pH sensing, spectroscopy, temperature sensing

## Abstract

Optical fibers have been used to probe various tissue properties such as temperature, pH, absorption, and scattering. Combining different sensing and imaging modalities within a single fiber allows for increased sensitivity without compromising the compactness of an optical fiber probe. A double-clad fiber (DCF) can sustain concurrent propagation modes (single-mode, through its core, and multimode, through an inner cladding), making DCFs ideally suited for multimodal approaches. This study provides a technological review of how DCFs are used to combine multiple sensing functionalities and imaging modalities. Specifically, we discuss the working principles of DCF-based sensors and relevant instrumentation as well as fiber probe designs and functionalization schemes. Secondly, we review different applications using a DCF-based probe to perform multifunctional sensing and multimodal bioimaging.

## 1. Introduction

From their compact size and alignment robustness, fiber optics-based sensing systems have become a powerful asset for a wide range of minimally invasive medical procedures to access organs deep inside the body [1,2]. A novel type of optical fibers—termed double-clad fiber (DCF)—is characterized by two concentric light guiding regions, the core and the inner cladding, and is extensively considered for multimodal bioimaging systems [3]. DCFs are now at the forefront of a new generation of multifunctional biosensors for performing various measurements while also providing imaging capabilities.

Fiber optics-based biosensors monitor biological or chemical reactions by combining a biochemical receptor with a detector relying entirely on optical transduction mechanism [4]. The receptor interacts with an analyte, or target biomolecule, to extract relevant information on the biological system. The transduction element, a DCF in forenamed biosensors, relays an optical signal to an external measurement unit. Modulations of the optical signal by analyte–receptor interactions, through either absorbance, reflectance, luminescence, scattering, or reflective index change [5], allows information collection. In most cases, the transducer is bidirectional, providing an excitation light to drive analyte–receptor interactions as well as collecting the optical signal generated by analyte–receptor interactions. A typical design will include a probe formed by the receptor and an optical element to manipulate the light coming in and out of an optical fiber end. The resulting probe is then inserted at the target site, normally deep inside biological tissue. DCF-based biosensors leverage the supplemental communication channel, inherent to the added cladding, to relay a combination of imaging information and biosensor measurements to an external acquisition unit, or excitation light to the target.

Reviews have been performed around fiber optics-based biosensors and their applications [6,7,8]; however, to our knowledge, no comprehensive reviews on DCF-based biosensors exist. This is despite a steady stream of research publications on DCF-based sensors. This review aims to fill this void by first covering the design of DCF-based biosensors and their functionalization to specific types of measurements. Combined sensing and multimodal imaging applications are thereafter reviewed. Finally, emerging technologies for future sensor designs and the constraints of bringing such novel sensors to the market are discussed.

## 2. Instrumentation

### 2.1. DCFs and Couplers

**Figure 1:**
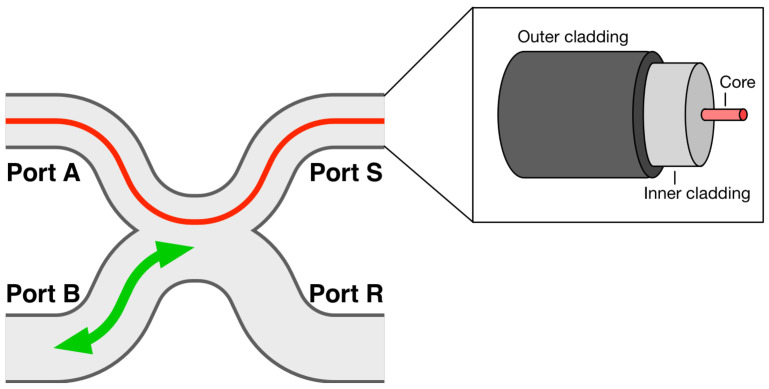
Schematics of a DCFC showing the single-mode signal in red and the direction of transfer of some multimode signals in green. Inset: concentric structures of a DCF.

A DCF is characterized by a concentric structure comprising two distinct guiding regions: a core and an inner cladding, as shown in the inset of Figure 1. Its core typically allows for the propagation of a single mode, associated with a given operation wavelength range, while its inner cladding can support the propagation of a multitude of modes, often hundreds. Such a fiber exploits the benefits of both single-mode and multimode propagation mechanisms. It enables either coherent illumination or detection through its core, or both, allowing, for example, for interferometric measurements. On the other hand, the multimode inner cladding can support a much higher power and benefits from a large numerical aperture (NA) and surface area, resulting in increased detection sensitivity. It can be used either for incoherent or partially coherent detection by carefully selecting its diameter for a given operation wavelength [9]. For incoherent imaging, switching the illumination between the cladding and core of the DCF allows for multiscale fiber-based sensing or imaging [10,11]. These features can be exploited for a variety of sensing applications and are especially suited for multifunctional sensors and multimode bioimaging systems.

To couple each signal in and out of the fiber with minimal losses, double-clad fiber couplers (DCFCs) have been introduced [12,13,14,15,16]. Figure 1 shows a DCFC in its most common and efficient embodiment, obtained by fusing a DCF to a second fiber, typically a multimode fiber. Such a coupler allows for the quasi-lossless transmission of a single-mode signal through the core of the DCF from a first port (Port A) to a second (Port S) and vice-versa. It also allows for the transfer of a multimode signal between the multimode fiber and the inner cladding of the DCF. In Figure 1, this corresponds to a signal transfer from Port B to the DCF’s inner cladding at Port S or vice-versa. The fourth port (Port R) is typically unused and can be packaged within the coupler to reduce the device’s return loss. A DCFC can either maximise the transfer from Port S to Port B, referred to as an “extraction” DCFC, or from Port B to Port S for an “injection” DCFC, or achieve transfer in both directions simultaneously, in a configuration referred to as a “bidirectional” DCFC [3].

### 2.2. DCF-Based Probe Design

Sensors are used to measure or detect localized signals. The intrinsic proximity between the sensor and the probed sample generally requires optical fibers for signal transduction. For diffuse or contact measurement, the fiber tip can be used as-is. This is common in oncological applications where fiber tips are put in direct contact with suspicious sites [17,18,19,20,21,22]. An optical system may be used at the fiber’s distal end to interface with the sample and optimize the measurement’s sensitivity. The primary goal of such an optical system is to manipulate the light beam to attain specific optical properties such as focal spot size, working distance, NA, or beam orientation. A radiometric-based model was proposed to evaluate probe designs and optimize simultaneously single-mode and multimode illuminations out of a DCF [23].

**Figure 2:**
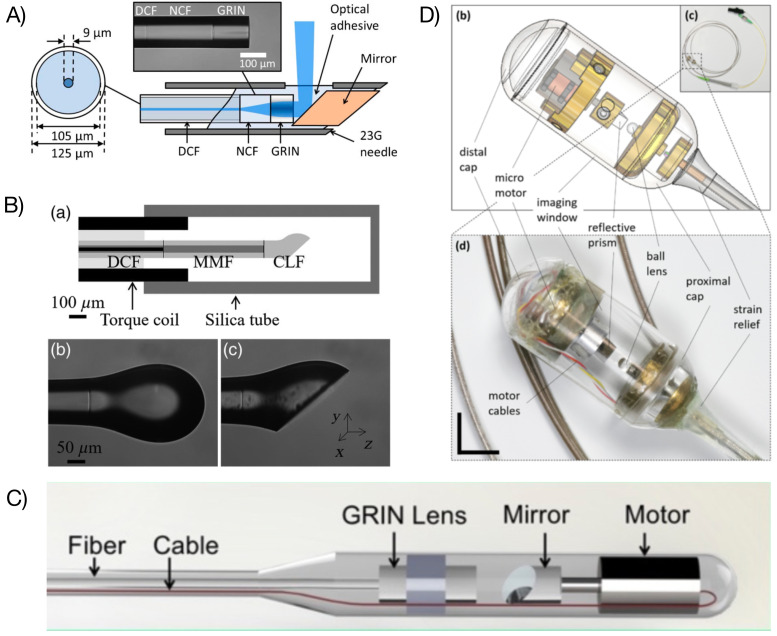
DCF-based probes and catheters. (**A**) Monolithic all-fiber needle probe [25]; (**B**) monolithic ball lens-based probe [41]; (**C**) micro-motor-based probe [36]; (**D**) capsule catheter [42]. Images reprinted with permissions from refs. [25,36,41,42]. Copyright 2013, 2019, 2021 Optica and 2020 SPIE.

A compact focusing system can be achieved either by using a monolithic design or a free-space arrangement using micro-optics elements. Figure 2 shows several probe configurations. A typical monolithic probe design consists of a glass spacer and a cylindrical lens. The spacer allows for beam expansion to provide a suitable NA. The beam is focused by the lens to the desired working distance and focal spot size. A tilted surface can be added to obtain a side-viewing configuration. Such designs can be achieved through bonding micro-elements, such as cylindrical rods and gradient-index (GRIN) lenses [24], using an all-fiber implementation by directly splicing the DCF to given lengths of no-core fiber (NCF) (or multimode fiber (MMF)) and GRIN fiber (Figure 2A) [25,26,27] or using a ball lens (polished or intact) that has been produced by melting a section of NCF (Figure 2B) [28,29,30]. A free-space arrangement using discrete micro-optics elements (Figure 2C) can also be used. This approach usually allows more freedom in selecting micro-optics elements to enable high NA or small focal spot, albeit at the cost of labor-intensive integration and alignment.

The optical system may also include some scanning mechanisms. When motorized or automatic beam scanning across the sample is required, two approaches are possible: proximal or distal scanning schemes [31]. Proximal scanning refers to the use of a motor proximally where the motion is relayed mechanically to the distal end of the fiber optics probe. This scanning pattern can be conveyed to the probe through back and forth radial movements or full rotations. A full rotation requires an interface between the fixed and moving parts of the system. For fiber optics probe, a rotary junction is typically used, consisting of a free space assembly transmitting the optical power from the fixed fiber end to the rotating fiber optics probe [26,32,33]. The rotational force is then transferred to the distal end of the probe via a mechanical shaft. While this approach allows for compact fiber optics probe ends, it sacrifices image sensitivity. The use of a rotary junction inherently comes at the cost of higher insertion loss as well as potentially generating ghost image artefacts [34]. Distal scanning involves the rotation of a reflector at the distal end of the fiber optics probe. Typical designs comprise a micro-mirror attached to the shaft of a micro-motor (Figure 2C) [35,36]. While this approach forgoes the use of a rotary junction, it impacts the overall size of the fiber optics probe. It also creates a shadow in the imaging field due to the electrical wires supplying the motor. A proposed design eliminates this shadow and reduces the footprint of the probe by anchoring the DCF to a concentric piezoelectric tube placed prior to the distal end of the fiber optic probe, with the latter acting as a cantilever. Applying a dephased waveform to two orthogonal electrode pairs forming the tube drives the probe in various spiral or Lissajous scanning patterns [37,38,39]. Leveraging the lower axial spread of two-photon microscopy using a DCF in conjunction with a miniature objective, shadow-free images can be obtained at the expense of a lower frame rate and the necessity to correct for angular lag at the probe end [40].

For in vivo or in situ measurements, an optical fiber probe needs to be packaged to preserve its integrity and biocompatibility. To reach organs through some inner lumen, such as the pulmonary system, the gastrointestinal tract, or arteries and veins, the probe may be inserted within a clear plastic tube [26]. Depending on the size of the studied organ, a fixation mechanism may be employed to reduce motion during the length of the exam and to center the probe within the lumen. For example, inflated balloons are often used for probing the esophagus [33,43]. More recent studies on imaging the upper gastrointestinal tract have introduced the use of a capsule (Figure 2D) [2,42]. The capsule contains the focusing and scanning optics, allows for self-placement of the catheter within the lumen, and enables the procedure to be performed on patients that are awake. For solid organs not accessible through a lumen, needle probes have been employed (Figure 2A) [25,44]. The fiber optics probe is inserted directly within needles as small as 25 gauge.

### 2.3. Fiber Optics Probe

Temperature, pH, displacement, distance, and pressure may be measured with a DCF-based probe.

#### 2.3.1. Temperature Sensing

**Figure 3:**
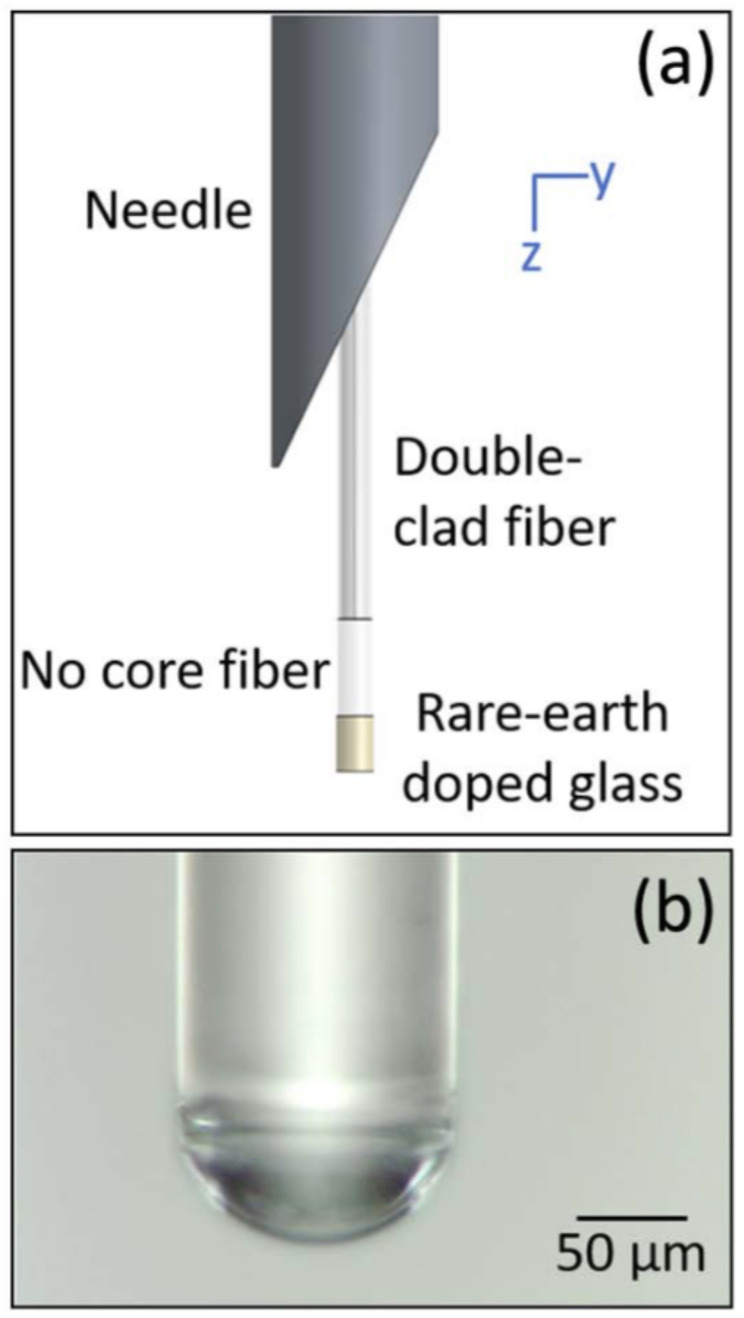
Needle probe for temperature sensing. Top (**a**): a DCF-based catheter coming out of a needle. Bottom (**b**): microscopic image of the probe tip with a curved focusing element. Images reprinted with permissions from ref. [44]. Copyright 2018 Optica.

DCF-based temperature sensing has been achieved by using rare-earth upconversion (Figure 3) [44]. The upconversion fluorescence emission from rare-earth ions—such as erbium—doped within a suitable host medium is related to temperature variations [45]. When the temperature of the host medium changes, ions undergo thermal excitation to a higher energy state, and the populations in two thermally linked states change [46]. Monitoring the ratio of fluorescence peaks from these two thermally linked states, which can be collected through the inner cladding of a DCF, allows ratiometric detection. As ratiometric measurement is independent of the excitation power through the DCF’s core or the concentration of rare-earth ions, this method is easier to implement with reliable results than fluorescence intensity-based methods.

#### 2.3.2. pH Measurement

**Figure 4:**
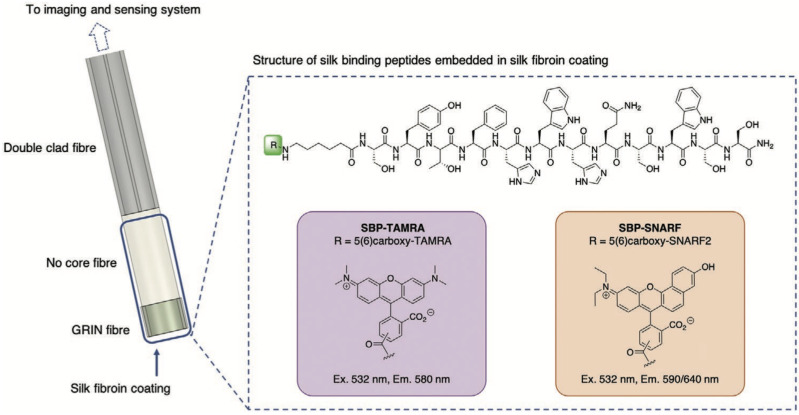
Silk-based functionalization of a DCF fiber probes for pH sensing. Image reprinted with permission from ref. [47]. Copyright 2021 Wiley-VCH GmbH.

DCF-based pH measurements have been realized by either injecting a pH-sensitive dye into some biological sample [27] or by attaching a pH sensor onto the fiber tip (as shown in Figure 4) [47]. The simplest method for pH detection via a DCF is by injecting the solution of a pH indicator into the biological tissue [27]. The fluorescence signal can then be detected by a DCF or a GRIN-NCF-DCF fiber assembly (as shown in Figure 2A). The excitation light travels through the core of DCF, and the emission light is collected through the inner cladding.

However, injecting a pH indicator into the human body may cause health issues and regulatory challenges. Recently, an alternative technique—attaching a pH sensor onto the fiber tip—has been proposed. pH sensing has not only been achieved at the tip of the solid fiber [47] but also along the micro-structured optical fiber (MOF) [48]. If a DCF-only or a GRIN-NCF-DCF fiber assembly is used [47], fluorescence tip sensing can also be performed by dip-coating a pH indicator onto the fiber tip or lens. Innovatively, a solid binding peptide (SBP) was recently utilized to covalently attach a pH indicator and avoid it from leaching into the biological tissue. As reported in Ref. [47], the fluorescent dye was washed away when SBP was not used. However, with SBP, the fluorescent dye retained even after an 18 h soaking period. If sensing over a distance is needed, a short section (e.g., 5–10 mm) of exposed core MOF can be dipcoated in the solution of a pH indicator to enable sensing along the entire length of the exposed MOF [48]. Khalid et al. [48] spliced a MOF onto a connectorized DCF to inject excitation light and collect emission light via a fiber-based setup.

Commercially available ratiometric pH indicators, such as seminaphthorhodafluor (SNARF) [27,47] or 5,6-carboxynapthofluorescein (CNF) [48], were utilized in previous DCF-based pH sensing studies. SNARF can be excited at 520–540 nm. It has two emission peaks (580–590 nm and 640 nm). With increasing pH, the 580–590 nm peak decreases, while the 640 nm peak increases. The ratio of these two peaks provides a reliable measurement of pH values. Similarly, CNF excites at 470–480 nm and has two emission peaks at 560 nm and 680 nm.

#### 2.3.3. Displacement, Distance and Pressure Sensing

Fiber optic-based displacement and distance sensors can be achieved through different approaches such as fiber grating-based sensing, Fabry–Perot interferometers, intensity-based sensing (including time-of-flight, triangulation, or confocal sensing), or low-coherence interferometers [49,50]. DCF-based distance sensors typically exploit the dual-channel feature of DCFs to achieve more compact and robust miniature probes. For intensity-based sensors, using the DCF’s core for launching and the inner cladding to collect the reflected light allows higher signal sensitivity and lower power fluctuations [51].

A simple dual-detection confocal displacement sensor can be achieved using a DCF in conjunction with a GRIN lens where the core and the inner cladding each acts as an independent detector [52]. Since the light coupling into the core comes from the focal plane while the inner cladding collects out-of-focus light, each channel acquires a different axial response. The ratio between each axial response provides an accurate displacement measurement. A plano-concave Fabry–Perot interferometric sensor fabricated on the end-face of a DCF has been demonstrated for ultrasound detection in photoacoustic imaging [53]. The sensor consists of a cavity formed by a pair of highly reflective mirrors, the first attached to the cleaved DCF tip while the second mirror is a spherical cap covering the Fabry–Perot cavity. The cavity is made by dipcoating the structure to form a polymer-based spacer. This sensor makes use of the DCF’s core to deliver a 1550 nm narrow-linewidth interrogation laser. In contrast, the inner cladding delivers a 650 nm multimode beam heating the cavity to tune the optical path length. The plano-concave Fabry–Perot cavity can also be formed by direct laser writing using two-photon 3D photolithography [54].

A similar strategy can be employed to construct a simple pressure sensor using a reflective deformable diaphragm across a DCF in a sealed cavity [55]. Pressure on the diaphragm will cause a slight deflection of the reflective surface and, thus, a variation of the inner cladding’s axial response. A simple pressure model can be inferred by injecting light in the core and taking the power ratio between injected light and light collected in the inner cladding of the DCF [55]. This type of sensor has the advantage of having a linear temperature–pressure relationship [56].

## 3. Applications

The use of a DCF allows the combination of different modalities through a single fiber. Typical implementations combine a structural imaging modality, such as optical coherence tomography (OCT) [57,58], with a complementary molecular or chemical sensing modality. OCT uses low-coherence interferometry to provide a refractive index-based structural contrast with a resolution of the order of tens of micrometers. However, utilization of a DCF comes at a certain cost on imaging quality as it may induce a multipath interference effect manifesting itself as ghost images [34]. Solutions to mitigate this effect have been proposed [33,59]. The section will review different applications highlighting the benefits of using a DCF for sensing purposes. Table 1 summarizes work combining more than one imaging modalities, while Table 2 repertories research combining imaging, sensing, and therapeutics by using a DCF.

### 3.1. OCT and Fluorescence-Based pH Measurement

Combined OCT and pH sensing can be attained using one single fiber: The OCT and fluorescence excitation light propagate through the core of DCF, and fluorescence emission is collected via the inner cladding of the DCF. It enables the co-localized and simultaneous acquisition of complementary information concerning structural changes and pH values in deep regions. In addition, the OCT function within such a biosensor allows for the visualization of the sample and precise placement of the biosensor. Due to the fact that the pH value within a biological sample can be highly heterogeneous [67], micrometer-sized fiber optic pH sensors need to be precisely positioned onto the region of interest to ensure accurate measurements [68]. Previously, image guidance for pH sensing was achieved by using a fiber bundle [69], which was unable to visualize tissue structures below the external surface of the sample. Instead, a DCF-based device can perform depth-resolved imaging via OCT. DCF-based OCT and pH measurements have been proposed to be used for in vitro fertilization (IVF) and cancer margin detection.

**Figure 5:**
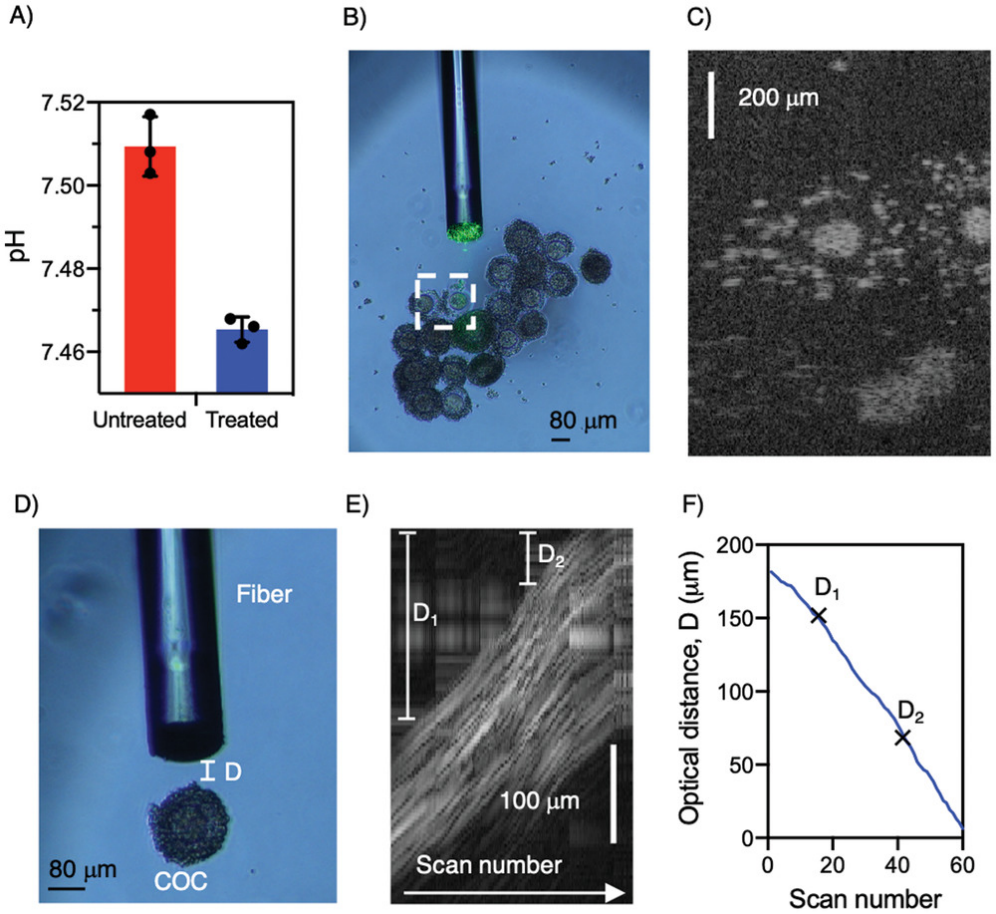
DCF-based OCT+pH probe for combined OCT and pH analysis of cumulus-oocyte complex. (**A**) pH measurements for untreated and ${\mathrm{COCl}}_{2}$ treated oocytes. (**B**) OCT+pH probe near oocytes with OCT field of view in white box. (**C**) OCT image of oocytes. (**D**) OCT+pH probe near single oocyte. (**E**) Composite OCT scan series of oocyte with decreasing optical distance D between probe and target. (**F**) Number of scans versus optical distance for composite image showcased in (**E**). Image reprinted with permissions from ref. [47]. Copyright 2021 Wiley-VCH GmbH.

IVF is a widely used alternative means of obtaining offspring for humans, livestock, and precious wildlife species [70]. Capon et al. [47] demonstrated the use of a combined OCT+pH biosensor for IVF application and showed that such a biosensor could be used to help collect oocytes from the follicles of an ovary (Figure 5). This DCF-based combined OCT+pH sensor uses OCT depth-resolved images to guide the accurate positioning of the aspiration needle to the oocyte (instead of towards a blood vessel or follicular fluid). It then measures the pH value, which is indicative of oocyte maturation [71].

OCT has been used for the detection of cancer margins [72] and lymph nodes [73], but its diagnostic accuracy is limited due to the lack of chemical contrast. Yet, it has been shown that cancer tissue exhibits lower extracellular pH [74]. Acting on this, Schartner et al. [75] developed a fiber-based pH probe and demonstrated its capacity to identify margins by using cancerous tissue from human breasts. Furthermore, Chen et al. [27] proposed that the combination of OCT and pH measurement by using a single DCF may enable accurate real-time detection of cancer margins, which is of great clinical significance: If malignant tumors are not eradicated, local cancer recurrence could happen. On the other hand, over resection, where both cancer and healthy tissues are removed, negatively affects local functional (e.g., removal of healthy brain tissue when treating brain cancer) and cosmetic outcome (e.g., removal of healthy breast tissue when treating breast cancer).

### 3.2. OCT and Fluorescence-Based Temperature Sensing

Similar to fluorescence-based pH sensing, DCFs can also allow combined OCT and temperature sensing via a miniaturized single fiber-based device. By using the core of DCF to collect the OCT signal, such a device can utilize the OCT function of the device to guide the precise placement of the associated temperature sensor [44]. This could be useful for temperature measurements deep inside the body (e.g., in a brain) and guiding temperature-based therapies. Temperature changes are affected by local physiological structures. For example, blood vessels take away heat such that measurements near blood vessels are not representative of the actual temperature of the region of interest [76]. Image-guided probe placement by using a DCF sensor may reduce measurement variations and the risk of damaging blood vessels [44].

### 3.3. OCT and Fluorescence-Based Molecular Sensing

DCF can be used to combine OCT imaging with fluorescence detection to probe specific molecular signatures of a sample. The DCF core is used for OCT while the inner cladding collects the fluorescence emission signal allowing an increased sensitivity. Either the core or the inner cladding can be used to deliver the fluorescence excitation light, although it was shown that a core delivery allows better fluorescence imaging resolutions [77]. Additional modalities can be added to such a system including red-blue-green (RGB) reflectance [63] or spectrally-encoded confocal microscopy (SECM) [78]. Fluorescence detection can be acquired over a large spectrum for spectroscopic analysis [12] or target a specific compound by detecting its associated wavelength band. These compounds can be exogenous, such as antibodies conjugated with fluorophores [79,80], or naturally occurring components associated with autofluorescence, such as nicotinamide adenine dinucleotide (NADH), flavins, or porphyrins [81].

A host of multimodal approaches have been investigated in the field of cardiology for atherosclerosis plaques characterization, including combined OCT and fluorescence sensing [82]. A proper assessment of the vulnerability of a plaque, which relies both on structural and chemical parameters, is critical to prevent plaque ruptures [79]. OCT can discriminate fibrous, lipid-rich, and calcified plaques as well as measure cap thickness while fluorescence sensing showed potential in detecting necrotic core lesions and inflammation, features that cannot be assessed by OCT alone [30,79]. The use of annexin V–conjugated Cy5.5 was investigated for the detection of necrotic cores and inflammation in a rabbit model [79]. Indocyanine green (ICG) was also shown to be able to target lipids and macrophages associated with inflamed vulnerable plaques, as was demonstrated in vivo in a rabbit model [29]. A time-gated fluorescence approach was also investigated to improve the molecular sensitivity and signal-to-noise ratio (SNR) of the system [83]. Autofluorescence can also be probed as necrotic cores have been shown to exhibit elevated autofluorescence signals in an ex vivo study on aortic cadaveric samples [30] and in an in vivo study on human patients [1]. Fluorescence lifetime imaging (FLIm), which assesses temporal dynamics of fluorescence emissions, has been investigated to detect features exhibiting structural and compositional contrast in human coronary artery [60]. By exploiting dynamic processes, FLIm can resolve fluorophores with overlapping spectra as well as discriminate the system-induced autofluorescence background from the sample signal. Necrotic cores have also been identified using frequency-domain fluorescence lifetime imaging, combined with OCT, by measuring the relative concentration of lipids and collagen on ex vivo cadaveric human coronary arteries [41].

Pulmonology can also benefit from a multimodal imaging approach using OCT to provide a structural contrast, while fluorescence sensing can probe the collagen and elastin fiber content of the tissue [77]. Lung imaging can be performed by using a needle probe [25] or a catheter inserted within a flexible bronchoscope [77]. An in vivo study on humans showed that while OCT can identify large vessels based on structural and speckle motion contrast, the autofluorescence signal can be used to map detailed vasculature network [77]. A combined OCT and autofluorescence system was used for needle biopsy placement guidance during lung cancer screening to increase the sensitivity of the procedure [62].

**Figure 6:**
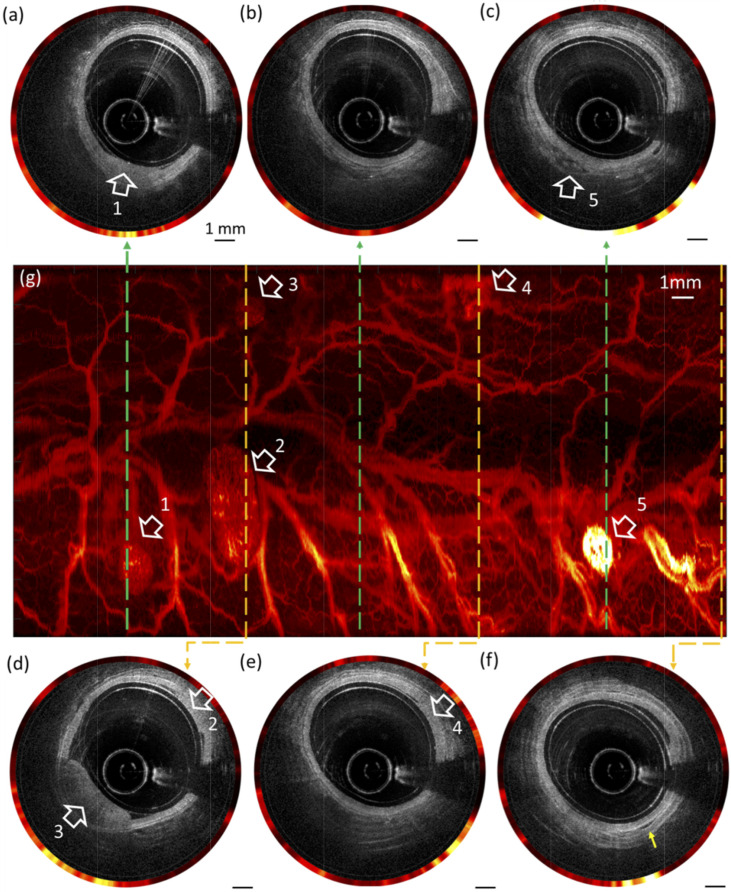
Combined OCT and fluorescence images (**a**–**f**) and en-face fluorescence image (**g**) of a colorectal wall from a rat model injected with ICG intravenously. White arrows show identified lesions. Image reprinted with permissions from ref. [36]. Copyright 2019 Optica.

Another vast domain of study exploiting the combination of OCT and fluorescence-based molecular sensing through a DCF is oncology, where such a system is used to identify pre-cancerous and cancerous lesions in various organs. Early cancer detection in the rectum can be achieved through the architectural and vasculature network assessment using ICG, allowing the identification and differentiation of normal and abnormal colons exhibiting hyperplastic polyp, adenomatous polyp, and adenocarcinoma [36,61]. Figure 6 shows the resulting images from a rat colon model labeled with ICG highlighting various lesions both visible on OCT and fluorescence images. Fluorescently labeled monoclonal antibodies, targeting specific membrane receptors, were used to study the tumor microenvironment and identify tumor masses in situ in a xenograft mouse model [35].

### 3.4. OCT and Reflectance-Based Spectroscopic Sensing

Molecular contrast can also be achieved by using reflectance-based spectroscopic sensing techniques. Similarly to fluorescence imaging, a DCF allows for the inclusion of a second modality for increased signal collection. A combination of OCT with hyperspectral imaging (HSI) using a DCF and DCFC has been demonstrated [84]. HSI allows the sample to be probed at multiple wavelength bands associating a spectrum to each acquired pixel. The authors demonstrated the capabilities of their system by imaging a healing epithelial wound where the OCT modality highlighted the topography of the sample while HSI provided an absorption spectrum of oxygen-saturated hemoglobin or an RGB projection. Attendu et al. [64] demonstrated a DCF-based system for combined OCT and multispectral imaging (MSI), similar to HSI but based on fewer wavelength bands. This could provide physicians with images comparable with white light video endoscopy (WLE), which is the standard of care in gastroenterology and pulmonology.

Single fiber reflectrometry (SFR) spectroscopy, where a broadband signal is emitted and collected through a single fiber [85], is another modality that can benefit from the use of a DCF-based system. SFR has been demonstrated in the fields of oncology [17,18,19,20,21], oxygen saturation monitoring [86], and orthopedics [87], for example. Attendu et al. proposed a multiscale OCT-SFR system based on a DCFC and a wideband multimode circulator (WMC) that allows the illumination to be switched between the core and cladding of the DCF port [11]. This multiscale system can either provide high resolution sensing or high SNR sensing, albeit with a lower resolution.

### 3.5. OCT and Multi-Photon Sensing

DCFs can likewise be exploited for multi-photon sensing such as multi-photon fluorescence and harmonic generation, where the single-mode core is used for the excitation and the cladding for collection. Since multi-photon generation processes benefit from intrinsic optical sectioning, the inner cladding for the emission collection does not affect the resolution [88]. Multi-photon sensing is typically used to probe endogenous molecules such as elastin or collagen [81]. A DCF may be used in multi-photon sensing alone to improve the SNR as no de-scanning is required [89]. DCFs have also been used to combine OCT and multi-photon sensing in the context of ovarian cancer [65]. This type of system enables concurrent two-photon and three-photon fluorescence, second-harmonic and third-harmonic generation, and OCT. Vega et al. [65] also presented a miniature multimodal objective making use of patterned dichroic surfaces with reflective optical power to create multiple optical paths in a single-lens system to accommodate different NA requirements associated with this multimodal system [90].

### 3.6. OCT and Reflectrometry

It is possible to use a multimodal approach to combine structural imaging with motion tracking. For example, El-Haddad et al. [66] demonstrated a system combining OCT, providing high-resolution imaging of the posterior retina and anterior chamber of the human eye, with spectrally encoded reflectometry (SER) for motion tracking. DCF-based SER allows partially coherent detection that increases collection efficiency and reduces speckle noise as well as an extended confocal parameter without sacrificing the lateral resolution resulting from single-mode illumination and multimode detection.

### 3.7. Structural Imaging and Distance Sensing

Previous sections relayed examples combining OCT, as a structural imaging modality propagating through the single-mode core, with a sensing modality. It is also possible to use the DCF’s inner cladding for intensity-based structural imaging, while the core can be used for interferometric distance sensing. Lemire-Renaud et al. [14] reported the combination of spectrally-encoded endoscopy (SEE) with interferometric depth detection based on a DCFC setup. SEE is achieved by illumination via the core and collection through the much larger inner cladding, allowing for higher sensitivities and lower speckle noise. Light collection through the core is sent to an interferometer to provide a height profile of the sample.

## 4. Perspective and Conclusions

In the past 15 years, DCF sensors have multiplied at an accelerating pace. Researchers have leveraged the benefits of using DCF sensors to demonstrate multifunctional and multimodal systems combining various imaging and sensing modalities. Compared to its single-mode counterpart, a DCF sensor enables dual propagation mechanisms without sacrificing the compactness of the fiber optics probe, increased sensitivity for most applications, or multiscale capabilities. However, most studies, as reviewed in this paper, are conducted to demonstrate the feasibility of utilizing DCF sensors. We believe that there are still many technical advances and biomedical applications to be explored and realized. Herein, several potential future directions will be discussed in more detail.

Emerging advanced manufacturing techniques, such as two-photon 3D photolithography, are likely to enable the physical fruition of novel DCF sensor designs to achieve unprecedented performances. For example, it has been proposed that a freeform micro lens-in-lens can be fabricated by two-photon 3D photolithography to allow an inner lens optimized for the core of the DCF and an outer lens optimized for the inner cladding of the DCF [91,92]. Such a freeform micro lens-in-lens has enabled a more than tenfold sensitivity boost while maintaining the compactness of the DCF sensor. This design is advantageous when light propagating through the core and the cladding requires different focusing optical configurations to acquire optimal measurements. This is often the case when combining OCT, a modality that needs a weakly focused beam, with a modality that prefers a high NA beam such as fluorescence-based sensing (Section 3.1, Section 3.2 and Section 3.3) or multi-photon sensing (Section 3.5).

In addition, the combination of DCF and advanced sensing techniques, such as microfluidics and microneedles-based devices, shows great potential. There have been a few promising attempts to use DCF to enable high collection/excitation efficiency for micro-flow cytometer [93] and surface-enhanced Raman scattering (SERS) sensing using a microfluidic chip [94]. In the cytometer study, high throughput (2500 particles/s) sensing was attained in part due to the use of DCF, which maximizes signal collection and reduces the reflection noise created at the fiber end-face [93]. Similarly, a depressed DCF has been demonstrated to enhance the interaction between the excitation light field and the sample in a SERS device [94]. Placed at different angles, DCFs may allow analysis of cell morphology [95]. Multiplexing sensing of different biochemicals, which are all excited and collected by a DCF, may become possible as detection techniques (e.g., microfluidic, time-gated, spectral-encoded, or spatial-resolved ones) continue to develop [96]. It can be expected that increasingly sophisticated sensing capabilities will be enabled with the use of dedicated DCFs. Pshenay-Severin et al. [97] used a custom double-core, double-clad, fiber with a monolithic GRIN lens-based probe design for multimodal nonlinear endomicroscopic imaging. Using co-registered coherent anti-Stokes Raman scattering, second harmonic generation, and two-photon excited fluorescence, they demonstrated label-free investigation of tissue structure, molecular composition, and correlation with function and disease status.

Most DCF sensors reviewed in Section 3 have been developed up to the prototype stage or a technology readiness level [98] below five, with the exception of systems reported in Refs. [1,99]. We expect to see wide adoptions of some DCF-based systems in the next years. Research translation and adoption not only balances the “technology push” from inventors (e.g., physicists, engineers, and chemists) but also the “demand pull” from potential users (e.g., biologists and clinical and industrial partners). This review aims to facilitate the research translation of DCF sensors by showcasing recent technical advances and applications to the potential users and highlighting opportunities for future development to both the users and inventors.

Beyond what has been reviewed in Section 3, several applications for DCF sensors are worth exploring. A potential clinical application of DCF-based sensors is for temperature-based therapies, such as radiofrequency ablation or laser thermal therapy. Cell death occurring during temperature-based therapies is closely related to accumulated thermal dose, which is dependent on both temperature increase and time of exposure [100]. Currently, these therapies suffer from high recurrence rates due to a lack of feedback [101]. For example, blood vessels, abundant in the liver, act as heat sinks and affect the uniformity and maximum temperature rise in the treatment regions. Although the dosage can be estimated using theoretical models, it is nearly impossible to take all effects, such as heat-sink effect or tissue heterogeneity, into consideration comprehensively. This can result in incomplete ablation or coagulation and potential increases in local tumor recurrence rates. To overcome this limitation, several strategies can be envisioned. Firstly, a combined OCT and temperature sensor may help by detecting blood vessels through DCF while simultaneously measuring real-time temperature rise. The effect of temperature could also be monitored indirectly by sensing variations in optical properties of the tissue [102,103]. Using a DCF would allow in this case the combination of OCT as a sensing mechanism with the delivery of a laser-based coagulation beam [34,104]. At higher adoption levels, DCF-based sensors will also play a remote monitoring role in hazardous atmospheres, where single-mode and multi-mode fibers already perform various sensing tasks [105,106]. In particular, they enable both single-mode and multimode propagation mechanisms through silica, a chemically inert material that maintains its shape under high-pressure [107].

In conclusion, DCF-based sensors have shown significant benefits for multifunctional sensing and multimodal bioimaging. As reviewed in this paper, this technology serves a wide variety of biomedical applications. As technology matures and additional tools are made available, it is expected that novel systems and applications will continue to emerge.

## Figures and Tables

**Table 1:** Summary of published applications for more than one DCF-based co-registered imaging modalities post 2016.

Combined Imaging Modalities	Results	Probe Design	Ref
Multiscale, Multispectral FLIm	Ex vivo imaging of tissue autofluorescence.	Monolithic all-fiber probe with GRIN lens	[10]
OCT + SFR	Improved morphological and molecular information imaging.	Benchtop reflective scanner head	[11]
OCT + NIRF	In situ imaging of a tumor in a xenograft mouse model of human colorectal cancer.	Micro-motor-based with GRIN lens	[35]
	In vivo colorectal cancer detection through identification and differentiation of normal colon and colon polyps.	Micro-motor-based with GRIN lens	[36]
OCT + FLIm	Ex vivo intravascular imaging and biochemical information capture at atherosclerotic plaques in arteries.	Monolithic ball lens- based probe with proximal rotary joint	[41]
	Ex vivo structural imaging and compositional contrast in unlabeled biological samples.	Monolithic all-fiber probe with GRIN lens	[60]
OCT + Fluorescence	Plug and play endomicroscopy system for gastrointestinal track imaging.	Tethered capsule with micro-motor and ball lens.	[42]
	High speed in vivo imaging of architectural morphology and vasculature of the rectum wall of a rat.	Micro-motor-based with GRIN lens	[61]
OCT + Autofluorescence	In vivo imaging of needle biopsy placement in lungs.	Monolithic needle probe with proximal rotary joint	[62]
OCT + Autofluorescence + SFR	In vivo sub-millimeter diameter probe for luminal organ imaging at 25 μm resolution.	Monolithic probe with rotary joint	[63]
OCT + MSI	Model and implementation of depth resolved spectrally sparse color imaging for deep organ structures.	All-fiber benchtop microscope	[64]
OCT + Reflectance + Multiphoton	High fidelity ex vivo tissue imaging.	Benchtop multimodal scanning optical microscope	[65]
OCT + SER	High-speed in vivo imaging of human retina at 2 gigapixels per second with micron spatial and millisecond temporal resolution.	Discrete optics benchtop microscope	[66]

**Table 2:** Summary of published applications for DCF-based co-registered imaging, sensing, or therapeutic modalities post 2016.

Imaging Modality	Second Modality	Results	Probe Design	Ref
OCT	Laser coagulation	Radiometric model for optimized imaging and sensing modalities in DCF probe.	Monolithic all-fiber needle probe with GRIN lens	[23]
OCT	pH	Ex vivo imaging and pH detection in biological tissue with accuracy of 0.01 pH unit.	Monolithic all-fiber needle probe with GRIN lens	[27]
		Imaging and pH change monitoring of lactic acid producing oocytes.	Monolithic all-fiber probe with GRIN lens and silk coating	[47]
OCT	Temperature	Ex vivo imaging and temperature sensing of rat brain.	Monolithic needle probe rare- earth doped glass lens.	[44]

## Data Availability

Not applicable.
